# Effect of Cell Age and Membrane Rigidity on Red Blood Cell Shape in Capillary Flow

**DOI:** 10.3390/cells12111529

**Published:** 2023-06-01

**Authors:** Mohammed Nouaman, Alexis Darras, Thomas John, Greta Simionato, Minke A. E. Rab, Richard van Wijk, Matthias W. Laschke, Lars Kaestner, Christian Wagner, Steffen M. Recktenwald

**Affiliations:** 1 Dynamics of Fluids, Department of Experimental Physics, Saarland University, 66123 Saarbrücken, Germany; 2 Institute for Clinical and Experimental Surgery, Saarland University, 66421 Homburg, Germany; 3 Central Diagnostic Laboratory-Research, University Medical Center Utrecht, Utrecht University, 3584 CX Utrecht, The Netherlands; 4 Department of Hematology, University Medical Center Utrecht, Utrecht University, 3584 CX Utrecht, The Netherlands; 5 Theoretical Medicine and Biosciences, Saarland University, 66421 Homburg, Germany; 6 Physics and Materials Science Research Unit, University of Luxembourg, L-1511 Luxembourg, Luxembourg

**Keywords:** red blood cells, erythrocyte, microfluidics, diamide, microcirculation, membrane rigidity, cell shape, density separation, red blood cell senescence

## Abstract

Blood flow in the microcirculatory system is crucially affected by intrinsic red blood cell (RBC) properties, such as their deformability. In the smallest vessels of this network, RBCs adapt their shapes to the flow conditions. Although it is known that the age of RBCs modifies their physical properties, such as increased cytosol viscosity and altered viscoelastic membrane properties, the evolution of their shape-adapting abilities during senescence remains unclear. In this study, we investigated the effect of RBC properties on the microcapillary in vitro flow behavior and their characteristic shapes in microfluidic channels. For this, we fractioned RBCs from healthy donors according to their age. Moreover, the membranes of fresh RBCs were chemically rigidified using diamide to study the effect of isolated graded-membrane rigidity. Our results show that a fraction of stable, asymmetric, off-centered slipper-like cells at high velocities decreases with increasing age or diamide concentration. However, while old cells form an enhanced number of stable symmetric croissants at the channel centerline, this shape class is suppressed for purely rigidified cells with diamide. Our study provides further knowledge about the distinct effects of age-related changes of intrinsic cell properties on the single-cell flow behavior of RBCs in confined flows due to inter-cellular age-related cell heterogeneity.

## 1. Introduction

**Figure 1:**
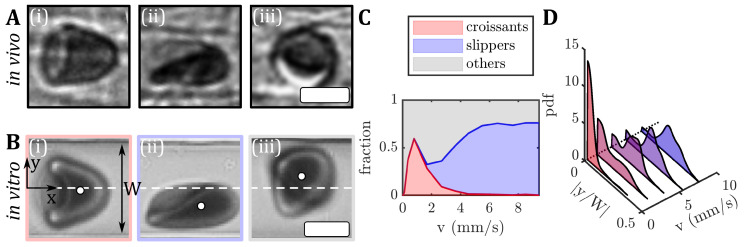
Images of RBC shapes in confined flows. (**A**) RBC shapes found in the mesentery of a hamster during in vivo flow. (**B**) Human RBCs flowing in a microfluidic channel: (**i**) centered croissant, (**ii**) off-centered slipper, and (**iii**) other shapes. The flow is from left to right. The white circles in (**B**) indicate the RBC center of mass and the white dashed lines indicate the channel centerline at y=0. The microfluidic channel has a width of approximately W=12µm and a height of H=8µm. The scale bars in (**A**,**B**) represent 5µm. (**C**) The shape phase diagram for RBCs in vitro from fresh blood of a healthy donor. (**D**) Probability density distributions (pdf) of the absolute values of the RBC *y*-position |y/W|, normalized by the microfluidic channel width *W* for different velocities.

Microvascular blood flow is vital for gas exchange and nutrient transport between blood and tissues. In the microvascular network, vessel diameters are similar to the red blood cell (RBC) size. At rest, healthy RBCs have biconcave, discocyte shapes with a diameter of roughly 8µm and a thickness of 2µm. They consist of a lipid bilayer membrane, a spectrin network on the inner surface, and the cytosol, which is rich in hemoglobin [1,2]. In the small vessels of the microvascular network, highly deformable RBCs flow in a single file and dynamically adapt their shapes to the vessel flow conditions [3,4,5], even passing through capillaries and apertures smaller than their own sizes [6,7]. Hence, various RBC shapes are found in vivo under physiological flow conditions (Figure 1A). In general, the RBC shape dynamics depend on external conditions, such as the vessel diameter and the flow rate, as well as intrinsic RBC properties, such as the membrane elasticity or the viscosity of the inner cytosol. Alterations in the RBC properties, such as impaired deformability, which is found in multiple diseases, such as malaria, diabetes, sickle cell disease, or neuroacanthocytosis syndrome [8,9,10,11,12], impact the RBC shape and impair blood flow and microvascular RBC transport [13,14]. However, the fundamental mechanisms through which such changes modify the RBC shape in microscale flows have not been characterized extensively.

Microscale RBC flow is commonly studied using microfluidic in vitro experiments [15,16,17,18,19,20,21], and numerical simulations [22,23,24,25,26,27,28]. In microscale single-cell flow environments, RBCs display a variety of stable and dynamic shapes, which depend on the channel confinement, flow velocity, the properties of the surrounding medium, and biophysical cell properties. Even under steady flow conditions, RBCs can exhibit various dynamical states, including snaking, tumbling, swinging, and tank-treading motions [14,29,30,31,32].

In rectangular microfluidic channels with dimensions similar to the RBC size, the shape complexity is shown to reduce to two dominant RBC shapes, namely the so-called croissant and slipper shapes (Figure 1(Bi,Bii)) [33]. Similar RBC shapes have been found in vivo in vessels of the human microcirculation [3] and in hamsters (see Figure 1A). In microfluidic channels, the symmetrically centered croissant shape predominantly appears at velocities below v<5mm/s, while the asymmetric off-centered slipper shape is found mainly at velocities above v>3mm/s. Consequently, a phase diagram of these main RBC shapes, as well as other shapes that do not exhibit stable modifications (Figure 1(Biii)), have been established to describe the occurrences of RBC shapes as functions of the flow rate in the microfluidic channel (Figure 1C) [33]. The resulting RBC shape in microcapillary flow is inherently coupled to its flow behavior, e.g., the cell’s equilibrium position in the channel cross-section. For healthy samples, croissant-like RBCs preferentially flow at the channel’s centerline at low velocities, whereas slippers flow closer to the channel’s side walls at higher velocities, resulting in pronounced peaks in the probability density distributions (pdf) at |y/W|≈0 and |y/W|≈0.22, respectively (Figure 1D). Deviations from the equilibrium RBC distributions have previously been used to assess changes in RBC flow behavior in patients with neuroacanthocytosis syndrome and COVID-19, as well as in patients before and after undergoing hemodiafiltration dialysis [34,35]. Moreover, RBC shapes in microfluidic capillary flow have been studied for healthy and diseased RBCs using manual and machine learning shape classification approaches [12,36,37,38,39,40]. Recent studies have shown the potential of microfluidic characterizations of RBC shapes as biomarkers for specific pathologies, to assess the cell deformability [34,41,42].

Nevertheless, fundamental knowledge about how the age of RBCs modifies their properties and, thus, affects their microcapillary flow, is still missing. RBCs have an average lifespan of 120 days in the circulatory system, after which, they are cleared by phagocytosis in the spleen based on their decreased deformability and other clearance mechanisms, which are still under debate [43,44,45,46]. During their lifetime, RBCs pass many times through tiny capillaries and traverse inter-endothelial slits, undergoing complex shape transitions and experiencing physical stress. This induces intrinsic physiochemical and morphological changes, such as a loss of surface area and volume, delayed shape recovery, and increased density and cytosol viscosity [47,48,49,50,51,52,53,54]. Numerical simulations generally show that changes in inner viscosity or the viscoelasticity of the membrane affect the RBC flow behavior [55,56,57]. However, experimental validation of how stable RBC shapes, even for healthy donors, are influenced by the cell’s age and corresponding RBC alterations remain scarce. Therefore, understanding the effect of age-induced changes in mechanical cell properties on the stable RBC shape signatures in microfluidic devices, which are used as fingerprints for in vitro microvascular flow assessment, is paramount.

In this study, we examine the flow of RBCs through rectangular microcapillaries. Specifically, we investigate the effect of RBC age and reduced membrane deformability on the stable RBC shape and the previously introduced shape phase diagram [33]. Although such rectangular channels do not capture the geometry of mostly circular in vivo vessels, they allow us to study RBC flow with similar cell shapes (see Figure 1A) and cell velocities as in the microvascular network, under controlled flow conditions with good optical access. For this, RBCs from healthy donors are fractioned according to age using density gradient centrifugation methods [58,59,60,61]. Furthermore, we treat fresh RBCs with a diamide at different concentrations to artificially increase the shear modulus of the RBC membrane and make the cells less deformable [5,62]. This enables us to determine to what extent rigidification of the cell membrane can be related to the changes in RBC shapes in flow during aging. In both groups, we observe distinct differences in the stable cell shapes during capillary flow and the fraction of overall stable shapes. Our study aims to advance our understanding of the flow behavior of RBCs in confined vessels. Mainly, how alterations of RBC biophysical properties, such as their deformability, affect microvascular blood flow.

## 2. Methods

### 2.1. In Vivo RBC Imaging

The in vivo images (Figure 1A) were obtained from experiments conducted on Syrian golden hamsters. These experiments were performed according to German legislation on the protection of animals and approved by the local governmental animal protection committee (permission number 25/2018). The animals were maintained on a standard 12/12 h day/night cycle, and water and food were provided ad libitum. The RBC flow in the hamster mesentery was captured using blue light intravital microscopic transillumination (Axio Examiner.D1, Carl Zeiss Microscopy GmbH, Göttingen, Germany), equipped with a high-speed camera (Hamamatsu Orca Flash 4.0, C13440, Hamamatsu Photonics Deutschland GmbH, Herrsching am Ammersee, Germany) [63].

### 2.2. In Vitro RBC Sample Preparation

Blood was collected into EDTA-containing tubes (1.6mg/mL EDTA, SARSTEDT, Nümbrecht, Germany) with informed consent from three healthy male voluntary donors (age 28–31 years). It was centrifuged for 5 min at 3000× *g* to separate RBCs and plasma. Sedimented RBCs were washed three times with a phosphate-buffered saline solution (Gibco PBS, Fisher Scientific, Schwerte, Germany). Finally, a hematocrit concentration of 1%Ht was adjusted in a PBS solution that contained 1g/L bovine serum albumin (BSA, Sigma-Aldrich, Taufkirchen, Germany). The viscosity of the PBS/BSA solutions at 20°C was approximately 1.2mPa s [56], similar to the viscosity of human blood plasma. Since we do not observe significant inter-individual variations in the results, data were averaged between the three donors.

Blood withdrawal, sample preparation, and microfluidic experiments were performed according to the guidelines of the Declaration of Helsinki and approved by the ethics committee of the “Ärztekammer des Saarlandes” (permission number 51/18).

#### 2.2.1. RBC Density Separation

**Figure 2:**
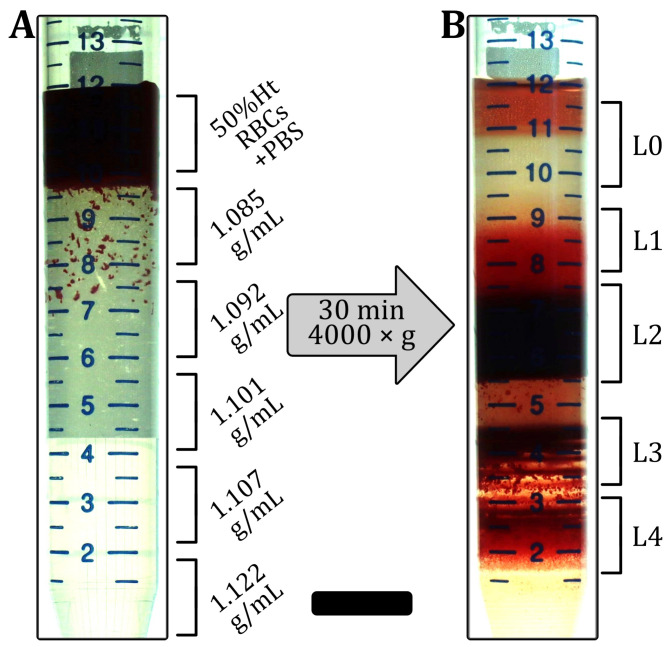
RBC density separation. (**A**) 2mL of 50%Ht-washed RBCs in PBS were placed on top of the five layers of the Percoll solutions. (**B**) After centrifugation, RBCs were fractionated into layers (L0–L4) with different densities. RBCs from L1–L4 were used for microfluidic measurements. The scale bar represents 10mm.

To fractionate RBCs based on their age, we performed Percoll density gradient centrifugation following the method described by Ermolinskiy et al. [60]. In brief, the Percoll solution (Cytiva 17-0891-01, Sigma-Aldrich, Taufkirchen, Germany), distilled water, and a 1.5M NaCl solution were mixed at five different ratios to obtain gradient solutions with different densities. Then, 2mL of washed RBCs with 50%Ht in PBS (ρ=1.025g/mL) were centrifuged on top of five layers of Percoll gradients with densities of 1.085g/mL, 1.092g/mL, 1.101g/mL, 1.107g/mL, and 1.122g/mL (Figure 2A). After centrifugation for 30 min at 4000× *g* at a temperature of 4 °C, four fractions of RBCs were obtained (Figure 2B) with the youngest cells in the top layers and the oldest cells in the bottom layer. Note that the different number of cells in each layer led to different shifts in the height of each layer. The topmost layer (L0 in Figure 2B) contained the PBS with mostly reticulocytes and leukocytes and was, therefore, not used in the microfluidic experiments. The other layers (L1–L4) were carefully extracted from the top to avoid any mixing. The fractionated RBCs were subsequently washed with PBS and resuspended at a hematocrit of 1%Ht in the PBS/BSA mixture.

#### 2.2.2. Membrane Rigidification

For the artificial membrane rigidification, fresh-washed RBCs were incubated in diamide (Sigma-Aldrich, Taufkirchen, Germany) for 30 min at diamide concentrations of 0mM, 0.5mM, 1mM, and 2mM. Diamide was proposed to induce a cross-linking between the spectrin proteins [64].

#### 2.2.3. Ektacytometry

The deformability of artificially rigidified RBCs and RBCs from the different density layers was evaluated using laser diffraction ektacytometry (Lorrca MaxSis, RR Mechatronics, Zwaag, the Netherlands). For this, the RBC samples were diluted to 0.5% in a PVP (polyvinylpyrrolidone) solution. The elongation index (EI), defined as EI=(a−b)/(a+b), where *a* and *b* represent the major and minor axes of the ellipse-shaped RBC diffraction pattern, respectively, is measured in a shear stress range of τ = 1–50 Pa at a temperature of $37{\;}^{{}^{\circ}}C$.

### 2.3. Microfluidic Setup

To assess the RBC shape in flow, we used rectangular microfluidic channels with a width of W=12.6±0.2µm, a height of H=7.7±0.2µm, and a total length of L=40mm. The microfluidic device was fabricated using polydimethylsiloxane (PDMS, RTV 615A/B, Momentive Performance Materials, Waterford, NY, USA) through standard soft lithography, which was bonded to a glass slide using a plasma cleaner (PDC-32G, Harrick Plasma, Ithaca, NY, USA). The inlet and outlet of the microfluidic chips were connected with rigid medical-grade polyethylene tubing (with an inner diameter of 0.86mm, Scientific Commodities, Lake Havasu City, AZ, USA) to the sample and waste containers, respectively. The microfluidic chip was mounted on an inverted microscope (Eclipse TE2000-S, Nikon, Melville, NY, USA), which was equipped with LED illumination (SOLIS-415C, Thorlabs Inc., Newton, NJ, USA).

We used a high-speed camera (Fastec HiSpec 2G, FASTEC Imaging, San Diego, CA, USA), and a 60× air objective (Plan Fluor, Nikon, Melville, NY, USA) with a numerical aperture NA=1.25; we imaged the RBC flow in the middle of the microfluidic chip at L/2. We used a high-precision pressure device (OB1-MK3, Elveflow, Paris, France) to apply constant pressure drops between *p* = 50 and 1000 mbar. A frame rate of up to 400 frames per second was used to record the image sequences of RBC passing the field of view. RBC shapes in flow were classified manually according to Guckenberger et al. [33]. All microfluidic experiments were performed at 22 °C. The center of mass of each cell in the projection plane was determined with a custom MATLAB (9.14.0.2206163 (R2023a), The MathWorks, Natick, MA, USA) algorithm, and individual cell velocities were determined by tracking the cell position over the image sequence within the field of view. In the applied pressure drop range, the resulting RBC velocity was between *v* = 0.5 and 9 mm/s (see Figure 1C). For the used microfluidic chip, we estimated the nominal wall shear rate in the straight channel as $\dot{\gamma }\approx 6v/H\approx$ 400–7000 s${}^{-1}$. Based on the viscosity of the surrounding medium of 1.2mPa s, we estimated the shear stress to be between τ≈ 0.5 and 8.4 Pa. This shear stress range is also probed in the ektacytometry measurements.

## 3. Results

### 3.1. Stable RBC Shapes in Straight Microchannels

**Figure 3:**
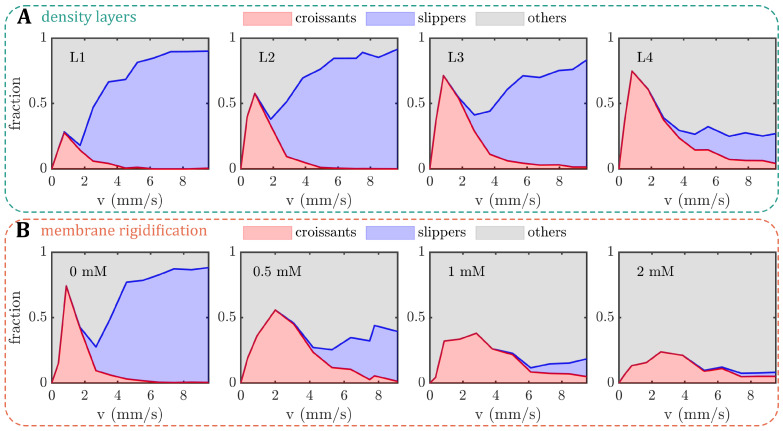
Shape phase diagrams (PDs) of RBCs in a microfluidic channel with a width of W=12µm and a height of H=8µm. The fractions of croissant-like, slipper-like, and other red blood cell (RBC) shapes are plotted as functions of cell velocity. (**A**) PDs for the different density layers (L1–L4) and (**B**) for different diamide concentrations. The analyses for (**A**,**B**) were performed on an average of 10,677 cells per donor (between 6361 and 14,268 cells) and 2871 cells per donor (between 1587 and 6168 cells), respectively.

Similar to the previously established phase diagram of stable RBC shapes (see Figure 1C), we examined the influence of the RBC age and artificially induced membrane rigidity on shape alterations within rectangular microchannels, focusing on the three dominant RBC shape classes, namely croissants, slippers, and others. The resulting cell velocities *v* in the microchannels are in the range of 0.5–9 mm/s, similar to the flow in the microvascular network [2,13]. For the density-fractioned cells, the number of croissant-shaped RBCs increases with increasing age (Figure 3A). While the peak value of the croissant fraction increases from roughly 25% to 75% from L1 to L4, the corresponding velocity of the croissant peak remains at approximately 1mm/s, in accordance with previous studies [33,37]. Furthermore, croissant-like shapes also appear at velocities v>5mm/s for L3 and L4, whereas no significant numbers of such shapes are found at the same velocities for L1 and L2. Concurrently, the amount of slipper-like shapes decreases with the increasing age between the layers. While a plateau value for the slipper fraction of roughly 80% is observed for v>6mm/s for L1 and L2, this value decreases sharply below 20% for L4. Additionally, the amount of other RBC shapes increases as the RBC age and density increase.

**Figure A1:**
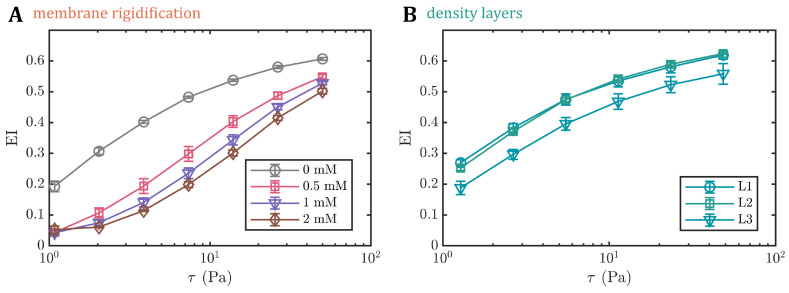
The elongation index (EI) (**A**) for different diamide concentrations and (**B**) for the different density layers (L1–L3) as a function of shear stress τ assessed through ektacytometry. For L4, no sufficient amount of RBCs was obtained for the ektacytometry measurements.

To artificially increase the shear modulus of the RBC membrane, we treated the cells with different concentrations of diamide and measured the elongation index (EI) by ektacytometry (Figure A1 in Appendix A). Within the range of 0.5–2 mM of diamide, we observe a dose-dependent decrease in deformability (Figure A1A). Note that the elongation index values of cells treated with diamide are lower than the EI values from the density-separated RBCs. For the layers, EI decreases with increasing age. Furthermore, the EI values from density-fractioned cells are similar to those of the control at 0mM (Figure A1B). Based on the EI measurements, we used the same RBC treatments for our microchannel approach. Increasing the membrane shear modulus also affects the formation of the stable croissant and slipper-like shapes (Figure 3B). While the control (0mM) exhibits the characteristic croissant peak at v≈1mm/s, increasing the diamide concentration leads to a reduction in the peak fraction and a shift of the peak position toward higher velocities of approximately 3mm/s for a concentration of 2mM. Furthermore, the croissant distribution in the shape phase diagrams broadens significantly, leading to the emergence of croissant-like shapes at v>5mm/s with an increasing diamide concentration. Simultaneously, the slipper plateau regime, which is observed at v>5mm/s for the control with 0mM, continuously decreases as the diamide concentration. At 2mM, the occurrence of slipper-like RBCs is ultimately suppressed, nearly completely, whereas most cells exhibit other shapes at high velocities.

**Figure A2:**
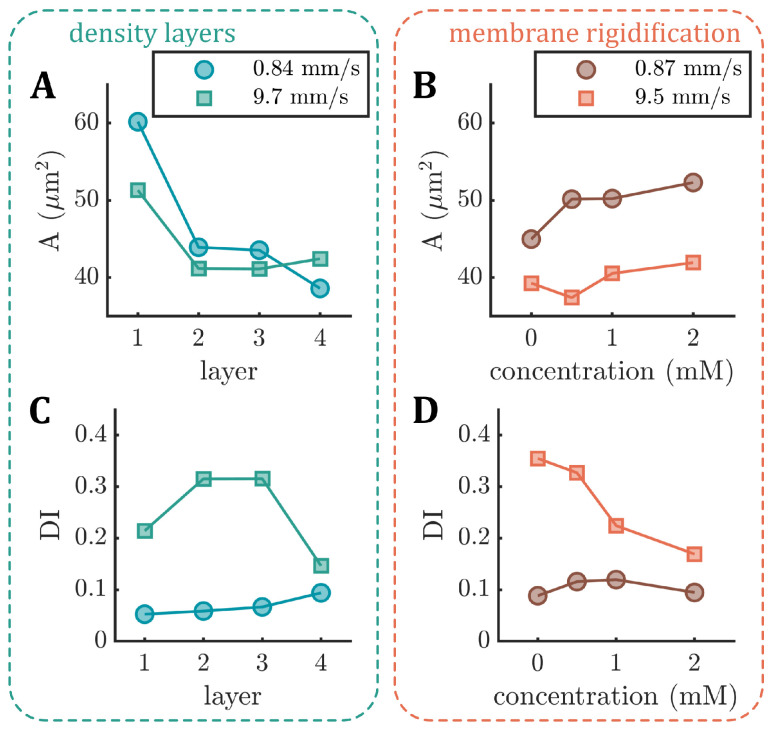
The median projection area (*A*) (top row) and median deformability index (DI) (bottom row) derived from the microfluidic measurements. (**A**,**C**) show the area and the deformability index for the different density layers (L1–L4). (**B**,**D**) show the area and the deformability index as functions of the diamide concentration. Data are shown for the representative average low (circles) and high (squares) velocities.

**Figure A3:**
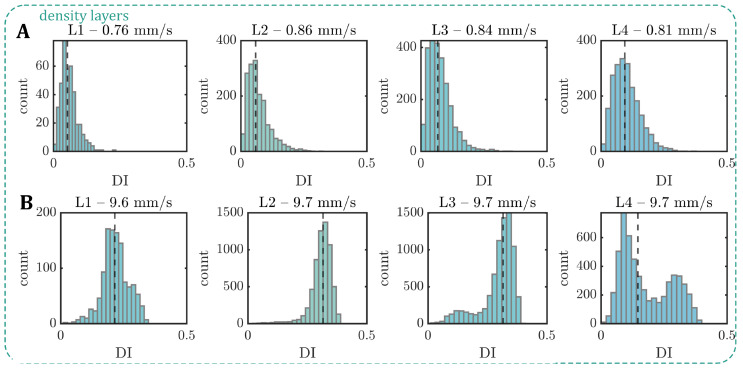
The deformability index (DI) derived from the microfluidic measurements for the different density layers (L1–L4) at (**A**) low and (**B**) high velocities. Vertical dashed lines correspond to the median DI for each distribution.

Based on the 2D projection of the RBCs in the *x*-*y*-plane of the microfluidic channel, we calculate the projection area *A* during the capillary flow. With the increasing age (L1–L4), the median projection area decreases at both low and high velocities (Figure A2A in Appendix A). In contrast, *A* increases with the increasing diamide concentration (Figure A2B). Note that although the volume and surface area of the RBC are coupled, an increase in the projection area does not necessarily correspond to an increase in the cell volume, i.e., when the RBC volume increases and its shape changes from a discocyte to a more spherical shape, its projection area can decrease. Moreover, we calculate the deformability index DI of the RBCs during the capillary flow, which is often used to assess changes in cell deformability [42]. Here, it is defined as DI=(a−b)/(a+b), where *a* and *b* are the major and minor axes of the RBC shape during flow. For both age-fractioned RBCs and cells treated with diamide, the median DI at high velocities is larger than at low velocities (Figure A2C,D) since the emergence of slipper-like shapes at elevated velocities leads to an elongation of the cell. While the deformability index does not significantly change at low velocities for both cases, we observe a drastic decrease in DI with the increasing diamide concentration at high velocities (Figure A2D). This effect is attributed to an increase in other shapes that emerge as the diamide concentration increases (see Figure 3B). These shapes often have more spherical, less elongated morphology (see Figure 1B) and, thus, a lower DI. Note that this decrease in the deformability index DI with the increasing diamide concentration assessed in the capillary flow is in accordance with the observed decrease in the elongation index EI from the ektacytometry measurements (see Figure A1). Moreover, for L4, we also observe a reduced median deformability index at high velocities. In this case, the occurrence of both other and slipper-like shapes leads to a pronounced double-peak profile in the DI distribution, which results in a decreased median DI (see Figure A3 in Appendix A).

### 3.2. RBC Equilibrium Position in the Channel Cross-Section

**Figure 4:**
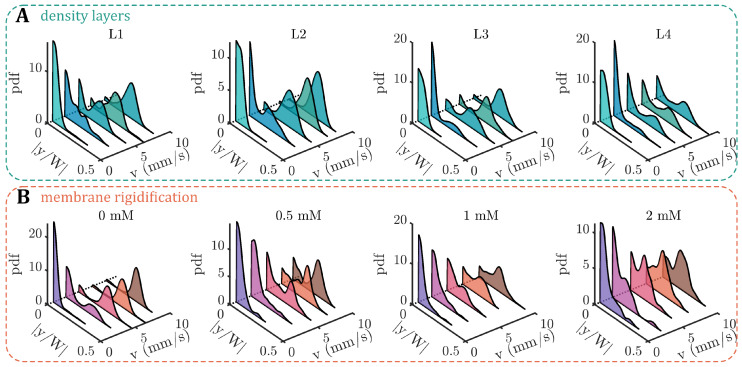
Probability density distributions of the absolute values of the RBC *y*-position normalized by the channel width |y/W|. Data are shown for five velocities for (**A**) the different density layers (L1–L4) and (**B**) diamide concentrations based on the data in Figure 3.

The RBC shape is inherently linked to its equilibrium position in the microchannel. While symmetric croissants generally flow at centered positions, asymmetric slippers emerge at off-centered positions with respect to the channel width *W*, as reported previously for fresh blood [33]. In the present study, the equilibrium position across the channel width is assessed based on the probability density distribution (pdf) of the absolute value of the normalized RBC *y*-position |y/W| as a function of the velocity (Figure 4). For all density-separated layers L1–L4, we observe a pronounced peak in the pdfs at the channel centers |y/W|=0 at low velocities v<3mm/s (Figure 4A). As the velocity increases, the central position is less favorable and an off-centered peak emerges at |y/W|≈0.22. For L1 to L3, this off-centered distribution corresponds to the shape transition toward slipper-like cells (see Figure 3A). In contrast, the pdfs at high velocities for L4 show broader distributions, comprising a second pronounced central peak, indicative of the croissant-like shape and other RBC shapes that emerge at v>5mm/s and |y/W|≈0 for L4.

Without the addition of diamide, the *y*-position distributions for fresh RBCs show the characteristic central or off-centered peaks at low or high velocities, respectively (0mM in Figure 4B). However, increasing the diamide concentration dramatically affects the equilibrium RBC position across the channel width. At a concentration of 0.5mM, we find a large number of cells that flow closer to the channel center, as evidenced by the emerging peak around |y/W|≈0 at high velocities. Simultaneously, the off-centered slipper peak is still visible, similar to the observations for L4. This effect qualitatively persists as the diamide concentration is further increased. At diamide concentrations above 0.5mM, more cells flow between the channel’s centerline and the off-centered position of the slipper peak, in accordance with the occurrence of other shapes beyond the stable croissants and slippers.

### 3.3. Fraction of Stable RBC Shapes

**Figure 5:**
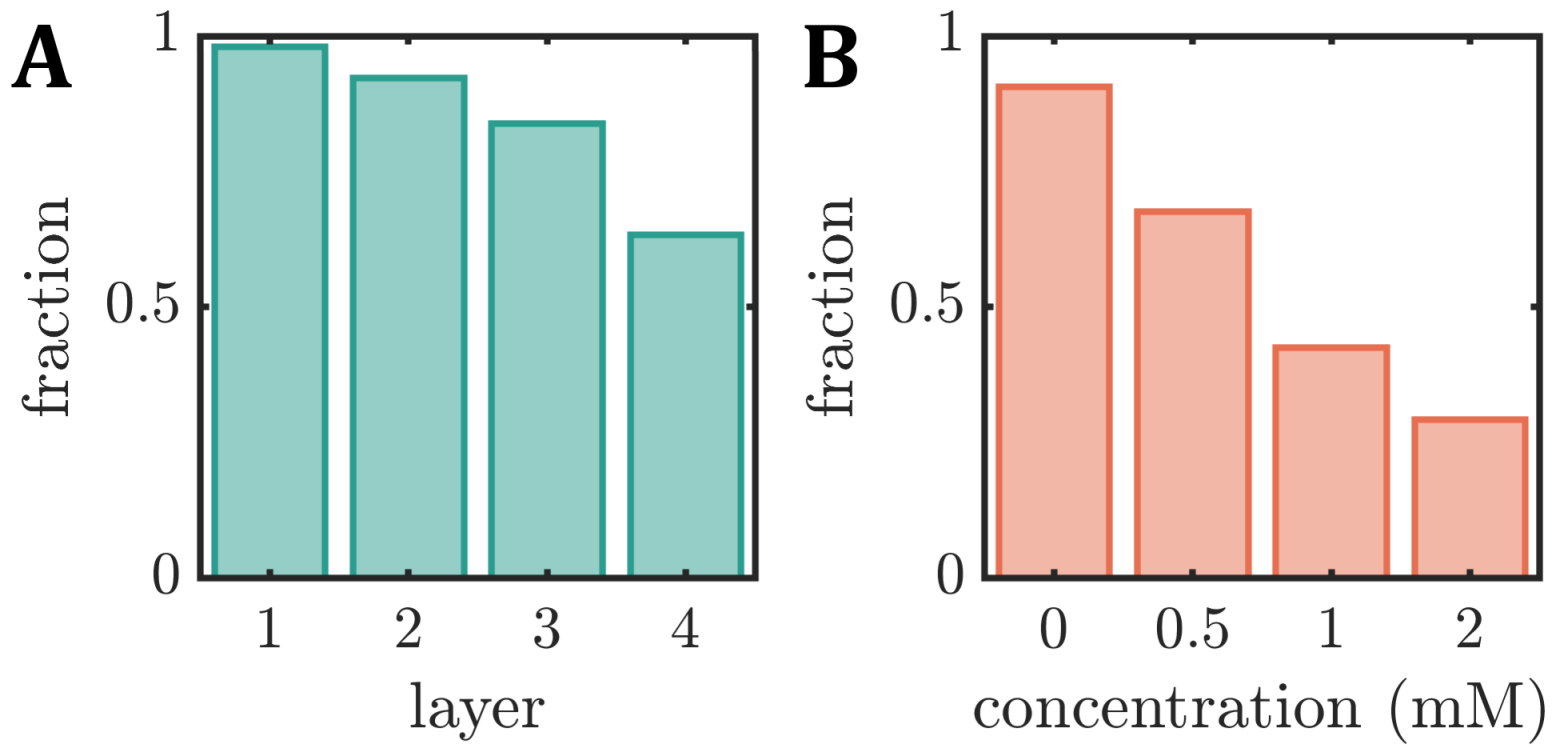
Fraction of stable RBC shapes, i.e., cells that do not rotate or tumble within the field of view during capillary flow, for (**A**) the different density layers (L1–L4) (**B**) as a function of the diamide concentration.

The changes in the RBC density and membrane rigidity affect the emergence of stable RBC shapes. Here, we define a stable shape when it does not rotate, tumble, or exhibit any other dynamic that significantly changes the cell’s *y*-position or shape within the field of view of approximately 300µm along the flow direction. Note that the field of view is 20mm downstream of the channel entry, which is long enough for any transient effects of the inlet to decay and achieve a stable shape configuration for all applied pressure drops [33,65]. In our study, the fraction of stable shapes decreases with the increasing RBC age for the density-fractioned cells (Figure 5A). While nearly all cells have the same shape in the field of view of L1, only 63% of the RBCs in L4 exhibit a stable shape. This decrease is in line with the increasing number of other shapes in the phase diagrams as the cells age (see Figure 3A). In the absence of diamide, roughly 90% of fresh RBCs exhibit stable shapes (Figure 5B), similar to the average value between L2 and L3. This fraction continuously decreases with the increasing diamide concentration, reaching 30% for cells treated with 2mM diamide.

## 4. Discussion

In this work, we study the effect of the RBC age and artificially induced membrane rigidity on the RBC shape in the flow setting, based on microfluidic experiments carried out on blood triplicates. We observe significant alterations in the RBC capillary flow behavior, specifically the stable RBC shapes, their equilibrium positions, and the fraction of stable shapes, as functions of both the RBC age and the different diamide concentrations. Foremost, the formation of slipper-like cells is continuously suppressed with the increasing age or diamide amount. Moreover, croissant-like RBC shapes start to emerge at higher velocities in this case. However, while the pronounced croissant peak corresponding to low velocities increases for old cells, it flattens down with the increasing diamide concentration. While we also observe changes in the deformability index and the projection area of the RBCs during the capillary flow, the shape phase diagram was recently shown to allow for a more precise evaluation of changes in RBC properties compared to such geometric parameters [40]. Note that L2 comprises the largest fraction of whole blood (see Figure 2B), with L3 being the second largest. Hence, the results of the control measurements without diamide (0mM) resemble the results of L2 and L3 regarding the phase diagram, the *y*-position distributions, the deformability index, the RBC projection area, and the fraction of stable shapes. This finding highlights the inter-cellular heterogeneity of fresh RBCs regarding their deformability. Characterizing sub-populations and individual cells has received increasing attention since such methods complement traditional hematological tests that rely on the average and mean values of RBC properties [66].

In general, the RBC shape in the microcapillary flow is primarily influenced by the intrinsic cell properties under otherwise fixed external conditions, i.e., velocity, channel geometry, confinement, and rheological properties of the surrounding fluid. The two main intrinsic parameters that were shown to influence the RBC dynamics in previous experimental and numerical studies are the cytosol and the properties of the plasma membrane. As the RBC ages, the viscosity of the cytosol increases, which was already suggested to suppress cell deformation, in general [6,49,67,68]. Consequently, the viscosity contrast λ between the viscosity of the cytosol and the surrounding fluid has been extensively studied, primarily using numerical simulation, which allows for a straightforward adjustment of λ. Although many studies use λ=1, it was shown that the viscosity contrast critically affects the flow of single vesicles and RBCs in the linear shear and Poiseuille flow [24,69,70,71,72,73,74,75,76,77,78].

In microfluidic experiments, the viscosity of the cytosol cannot be determined and changed straightforwardly. Nevertheless, the viscosity contrast can be tuned by changing the outer viscosity by using dextran solutions. Recent experiments in rectangular microchannels with a diameter similar to the RBC size demonstrated that decreasing the viscosity contrast λ results in the emergence of slipper-like RBCs at lower cell velocities [56]. This is in qualitative agreement with our observations that with a decreasing RBC density, hence, decreasing λ, slippers are the predominant shape (see Figure 3A). As λ increases with increasing age, the fraction of highly deformed, asymmetric, slipper-like RBCs decreases. Simultaneously, the occurrence of centered symmetric croissants appears to be enhanced by a higher inner viscosity, as indicated by the strong peak for L4.

Nevertheless, not only do the properties of the cytosol change as the RBC ages, but also the properties of the cell membrane, including the elastic shear modulus and membrane viscosity [47]. For healthy RBCs, the cell membrane exhibits viscoelastic properties and deforms at a constant surface area during the flow. In numerical simulations, the RBC membrane and the mechanical properties of the spectrin cytoskeleton are, therefore, often modeled as a two-dimensional elastic membrane with resistance to shear and area dilation, as well as resistance to bending [79,80,81], which allows capturing dynamic and steady RBC shapes [24,27,41,71,76,82,83,84].

The effect of an artificial increase in the shear modulus of the RBC membrane is clearly visible in our microfluidic experiments (see Figure 3B). Early micropipette aspiration experiments already highlighted that the deformation of RBCs depends on the viscous and elastic properties of the cell membrane [85,86]. These concepts were integrated into numerical simulations, which recently showed that the microcapillary RBC dynamics are crucially affected by the characteristics of the cell membrane, whether it is modeled as purely elastic or viscoelastic [55,57,87]. Gürbüz et al. [57] found that the RBC shapes with membrane viscoelasticity resemble the experimentally observed shapes during the start-up in a 10µm capillary [88]. Although both approaches with and without the incorporation of a membrane viscosity result in the formation of centered symmetric cell shapes, the shape transition time and the exact RBC deformation patterns are significantly influenced by increasing the membrane viscoelasticity. Based on the data of Gürbüz et al. [57], the resulting stable cell shapes with increased membrane viscoelasticity would be classified as others in our work. Thus, we hypothesize that increasing the viscoelastic properties of the RBC membrane results in the suppression of highly deformed slipper cells at high velocities in combination with the emergence of other non-stable cells, in accordance with the observations of L4 and an increasing diamide concentration (see Figure 3). Based on the assumption that with the increasing RBC age, the membrane rigidity increases, we note that the phase diagram of L4 resembles the one of a diamide concentration of 0.5mM. However, while increasing the amount of diamide further suppresses slipper-like cells, it also decreases the number of croissants at low velocities. This indicates that the microcapillary flow behavior of older RBCs is indeed governed by both an increase in the membrane rigidity and an increase in the cytosol viscosity.

## 5. Conclusions

To conclude, our results demonstrate the sensitivity of the RBC shape on their age-induced intrinsic properties, highlighting the importance of considering the heterogeneity of cell populations in microfluidic deformability assessments or diagnostic applications. Moreover, regarding the investigations on in vivo aged cells, we show that the artificial rigidification of the membrane with a low diamide concentration (0.5mM) resembles the microcapillary flow behavior of the oldest RBC fraction the most. In vivo, the entire situation is more complex, because, in addition to the RBC properties, the conditions in the microcapillaries are less well-defined. This starts with the geometry of the vessels, which are different from the microfluidic channels, and even diameters may be subject to temporal alterations due to vasodilation and vasoconstriction. This continues with a varying flow speed due to a changing pulse rate and blood pressure. Finally, the physiological and pathological variations of the blood plasma composition alter the biophysical effects (λ) as well as the biochemical effects on RBC flow properties [35]. Therefore, our study presents an initial methodological in vitro framework for future experimental and numerical investigations of how alterations in RBC properties influence their shape in capillary flow at the single-cell level. This research is highly relevant for the accurate simulations of RBCs and the in silico modeling of blood flow.

## Data Availability

All relevant data are included in the article, further inquiries can be directed to the corresponding author. Source data files for the figures are provided in the supplementary material file.

## Supplementary Materials

The following supporting information can be downloaded at: https://www.mdpi.com/article/10.3390/cells12111529/s1, Table S1: Source data files for the figures.

## Abbreviations

The following abbreviations are used in this manuscript:

BSA	bovine serum albumin
EI	elongation index
Ht	hematocrit
L*x*	layer no. *x*
PBS	phosphate-buffered saline
PD	phase diagram
pdf	probability density distribution
PDMS	polydimethylsiloxane
RBC	red blood cell
